# Literary Notes

**Published:** 1902-01

**Authors:** 


### LITERARY NOTES.

*The Homeopathic Journal of Pediatrics*, is the title of a new medical journal to be published in Buffalo on or about January 1, 1902. This will be the only journal in this country, in the Homeopathic School, that is devoted entirely to diseases of children. The subscription price will be $1.00 per annum, and the journal will appear each month. The editor is Dr. J. G. Chadwick, of 382 Franklin Street. We wish the new enterprise from its beginning an uninterrupted success.

THE *Journal of the Association of Military Surgeons* has made its appearance. It is to be published quarterly, and takes the place of the annual volume of Transactions. The two numbers already issued present a fine appearance. The journal is edited by the secretary of the Association, Captain James Evelyn Pilcher, M.D., passed assistant surgeon U. S. Army (retired) and is printed at Carlisle, Pa.

THE *Western Reserve University Bulletin* is issued quarterly. The fourth number of Volume VI., November, 1901, contains addresses delivered at the seventy-fifth anniversary commencement day, '01, by some prominent men, a dietary of studies, an account of commencement week, and notes by the editor. Price, 50 cents.

THE Thirtieth annual report of the Buffalo State Hospital has been received. It is for the year ending September 30, 1900, and the hospital then contained 1,880 patients. The crowded state of even the much enlarged hospital, has necessitated the recent transfer of a large number of patients to Willard State Hospital. The report indicates the continued prosperity of the hospital under the able superintendency of Dr. A. W. Hurd.

